# Thyroid hemiagenesis with a TI-RADS 2 nodule in the contralateral lobe

**DOI:** 10.1186/s13044-021-00101-5

**Published:** 2021-04-30

**Authors:** Senai Goitom Sereke, Anthony Oriekot, Felix Bongomin

**Affiliations:** 1 Department of Radiology and Radiotherapy, School of Medicine, Makerere University College of Health Sciences, Kampala, Uganda; 2 Department of Medicine, School of Medicine, Makerere University College of Health Sciences, Kampala, Uganda; 3 Department of Medical Microbiology and Immunology, Faculty of Medicine, Gulu University, Gulu, Uganda

**Keywords:** Hemiagenesis, Thyroid, Neck ultrasound, Nodule, TI-RADS

## Abstract

**Background:**

Thyroid hemiagenesis is a rare congenital anomaly in which one lobe of the thyroid gland fails to develop. There is an increased incidence of associated thyroid disorders in patients with thyroid hemiagenesis.

**Case presentation:**

A 32-year-old Ugandan woman presented with a complaint of painless neck swelling of 3-months duration. The swelling was associated with a globus sensation. There was no history of thyroid – related problems or treatment prior to this presentation. Physical examination demonstrated a mobile right thyroid swelling without an obvious nodular contour. Neck ultrasound showed an absent left lobe of thyroid gland, a right lobe with a solitary nodule scoring two points on the Thyroid Imaging, Reporting and Data System (TI-RADS) and an isthmus *in situ*. Extensive search for possible ectopic thyroid tissue was negative. She was biochemically euthyroid. The patient was counseled about thyroid hemiagenesis and was put on a regular follow up in the clinic for the TI-RADS 2 nodule.

**Conclusion:**

Thyroid hemiagenesis is often associated with other thyroid disorders. Its diagnosis should prompt an active search for other associated morphological or functional thyroid abnormalities.

## Background

Thyroid hemiagenesis (THA) is a rare congenital disorder that is characterized by an absence of one thyroid lobe with an estimated prevalence rate of about 0.02 % [1]. This anomaly is often detected incidentally. The pathogenesis and clinical significance of this malformation remain unclear. Hence there is no specific recommendation for management, especially in asymptomatic cases [1, 2].

The underlying mechanisms in thyroid morphogenesis or agenesis is poorly understood [3]. Congenital thyroid anomalies may be caused by either an abnormal descent of the gland or by incomplete genesis of a lobe. However, the etiology still remains unclear [4]. Genetic aberrations may have a role in the etiology of this disorder, as reported in monozygotic twin studies [5].

THA may involve either lobe, with or without agenesis of the isthmus. Studies carried out in living population showed that it affects the left lobe in 80 % of the cases (with left to right ratio  of 4:1) [6]. Left lobe hemiagenesis is associated with agenesis of the isthmus in 50 % of cases while right lobe agenesis is predominantly associated with isthmus agenesis [7].

Patients with THA are most frequently clinically euthyroid and will have normal circulating levels of thyroxine (T4) and triiodothyronine (T3). If THA is suspected clinically, the diagnosis can be confirmed by imaging techniques, such as ultrasonography or thyroid scintiscan [8, 9]. Less frequently, the anomaly may be detected incidentally on cross-sectional imaging performed in the evaluation of other medical conditions. The utility and popularity of thyroid ultrasonography has grown to almost gold-standard status due to its wide availability, non-invasiveness, and low cost [9].

 Herein, we present a case of an adult Ugandan woman in whom we diagnosed a biochemically euthyroid left THA. To the best of our knowledge, there is no record of a published case of THA from a sub-Saharan African country.

## Case presentation

A 32-year-old Ugandan woman with an unremarkable past medical history presented with a 3-month history of right sided neck swelling. The swelling was associated with an occasional localised pain and sensation of an object or food stuck in the throat (*globus* sensation). There was no documented treatment related to the thyroid gland, or a history of head and neck surgery. She reported no known family history of thyroid – related problems. She had no history of menstrual irregularities and had given birth to two children – both of whom are alive and healthy.

Physical examination demonstrated a right-sided mobile thyroid swelling without any obvious nodular contour. Her blood pressure was normal at 90/60mmHg with a normal pulse rate of 80 beats per minute. Auscultation of the swelling was unremarkable without any bruit.

**Fig. 1:**
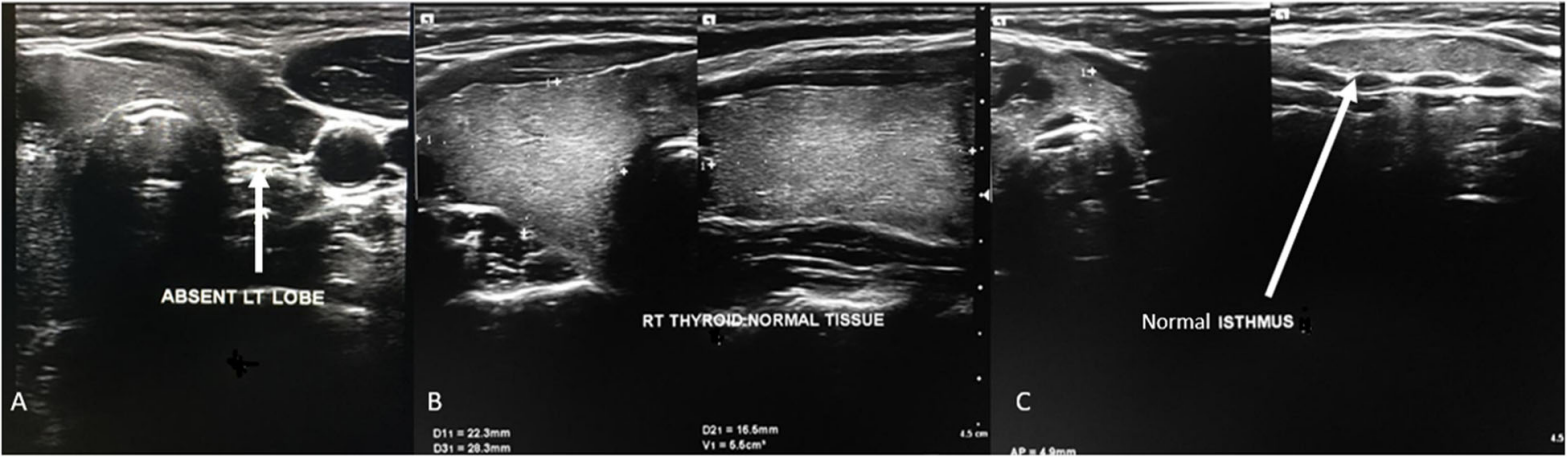
Ultrasound of the thyroid gland with high frequency linear probe  demonstrating **a** Absent left thyroid lobe **b** The right thyroid lobe normal tissue volume, and  **c** Isthmus in situ both in longitudinal and transverse planes

**Fig. 2:**
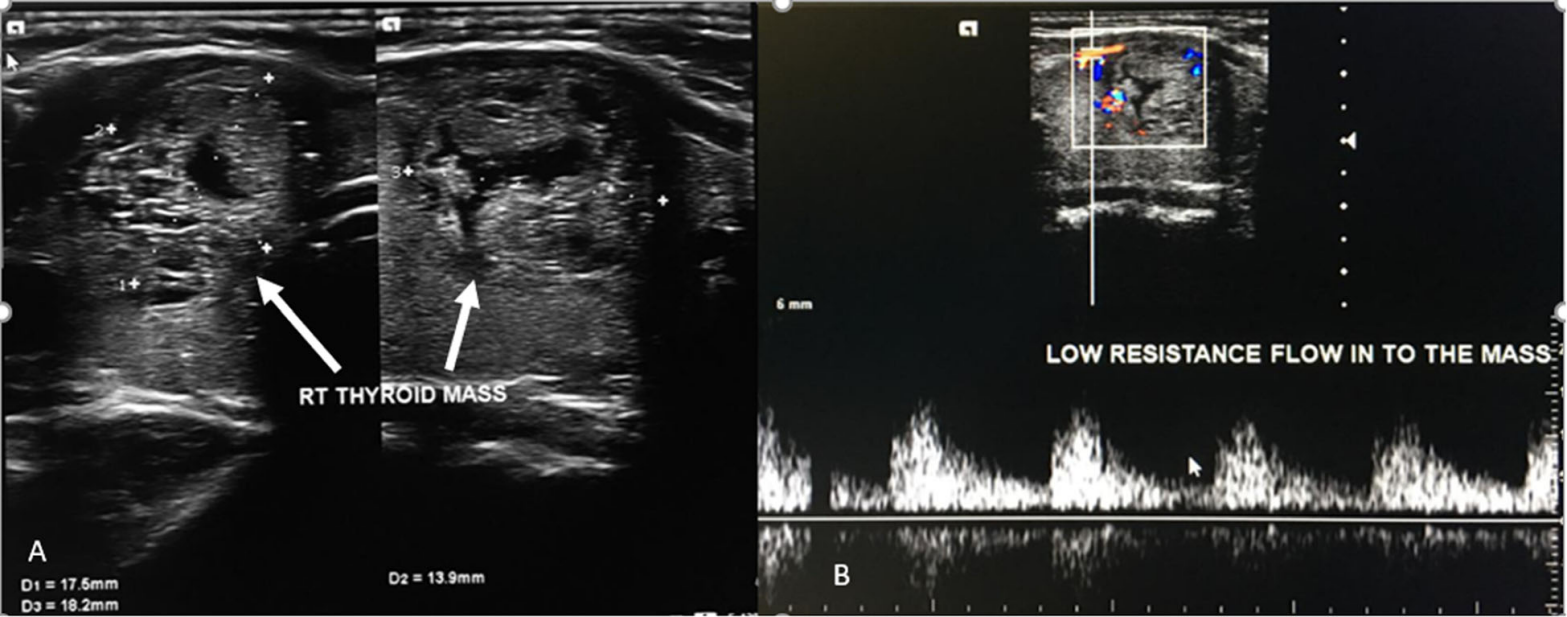
Ultrasound of the right thyroid nodule **a** a well-defined nodule, with smooth margins, wider than taller, hypoechoic, spongiform composition, and with no echogenic foci **b** Color and spectral Doppler (triplex Doppler) of the nodule showing moderate and low resistance flow respectively

Ultrasound examination of the neck showed a well-defined nodule in the right lobe of thyroid gland, measuring 1.8x1.8x1.4cm. According to the American College of Radiology, Thyroid Imaging, Reporting and Data System (TI-RADS) categorization, it had smooth margins (point 0), wider than taller (0), hypoechoic (2), spongiform composition (0), and with no echogenic foci (0) It had mild to moderate flow on color Doppler and low resistance flow on spectral Doppler. There was no left lobe of the thyroid, but the isthmus of the thyroid was in *situ* and measured 0.5 cm in antero-posterior diameter. The remaining normal right lobe of thyroid measured 2.2 × 2.8 × 1.7 cm (Vol 5.5ml) (Figs. 1 and 2). There were no enlarged cervical lymph nodes. The ultrasound findings prompted the examiner to search for any possible ectopic thyroid tissue. A thorough upper neck ultrasound examination found no thyroid tissue in the suprahyoid, prehyoid, infrahyoid, submandibular, sublingual and prelaryngeal region. The ultrasound findings were therefore consistent with the diagnosis of a right thyroid lobe nodule (TI-RADS 2) and left thyroid lobe hemiagenesis.

The serum thyroid stimulating hormone (TSH) level was 3.92 µIU/ml (reference range 0.27–4.20), Free T3 3.21 pmol/L (3.10–6.80) and free T4 11.79 pmol/L (12.0–22.0).

The patient was counseled about her condition and  offered for a regular follow up at the endocrinology clinic for her TI-RADS 2 nodule.

## Discussion

THA is a rare congenital anomaly of the thyroid gland with about 800 cases reported in the literature until 2020 [5]. The true prevalence of THA is not clearly known, with the reported prevalence rates varying between 0.05 to 0.5 % [1]. In this report, we present a case of woman diagnosed with left lobe THA without involvement of the isthmus and a right-sided thyroid nodule (TI-RADS 2) on ultrasound. In Uganda, since the introduction of the universal iodization in 1993, iodine fortification of salts has markedly reduced the incidence of goiter [10]. Therefore, it is important than all suspicious lesions are meticulously investigated. In the present case, we cannot completely rule out the possibility of early childhood exposure to iodine deficiency since she was born before the era of universal salt iodization in Uganda.

Patients with THA are most frequently clinically euthyroid as in the present case [8]. However, large case-control studies observed a significantly higher incidence of concomitant thyroid disorders such as Gravesʼ disease, Hashimotoʼs thyroiditis, subacute thyroiditis, nodular goiter, hyper – functioning adenoma, primary carcinoma, and metastatic carcinoma, in patients with THA than in those with bilobate thyroid glands, the most frequent disorders being thyroid nodules and autoimmune thyroid diseases [8, 11]. We found a solitary thyroid nodule in the present case of THA as a reason to seek medical attention.

Most cases of THA are sporadic, however, familial clustering of THA has also been reported [1, 12]. The prime genes mentioned in association with THA include thyroid transcription factors – TTF1 (NKX2–1), TTF2 (FOXE1) and PAX8. Homozygous deletions of one of these genes in animal models led to thyroid dysgenesis of varying severity [1, 13]. The impact of THA on the patient’s general health is not clear. Nonetheless multiple associated conditions have been described in the literature, of which parathyroid abnormalities were the most common [1, 14].

Ultrasonography and radionuclide thyroid scanning are the imaging modalities of choice in the evaluation of the thyroid gland [15]. Thyroid scintigraphy using technetium or iodine can be helpful in the diagnosis of THA but has drawbacks due to artefacts related to non-visualization of one thyroid lobe due to neoplasm, contralateral autonomous solitary thyroid nodule that is suppressing normal tissue, and inflammatory; and infiltrative diseases of the thyroid [16, 17]. Therefore, scintigraphy findings should be confirmed by ultrasound to avoid false positive results [18]. Ultrasonography is a better diagnostic tool as it is widely available and cost-effective with no radiation exposure to the patient [19]. However, in case of ectopic thyroid gland in the retrosternal or mediastinal location, scintigraphy with I-123 is the investigation of choice as it has high specificity [20]. In our patient, the diagnosis of THA was confirmed with ultrasound of the neck.

TI-RADS lexicon was developed by the American College of Radiology for risk stratification of a thyroid nodule using ultrasound to guide further management [21]. Stratification is based on composition, echogenicity, shape, margin, and echogenic foci of the thyroid nodule. According to the current recommendations, TR1 – benign (no fine needle aspiration (FNA)), TR2 – not suspicious (no FNA), TR3 – mildly suspicious (FNA if ≥ 2.5 cm, follow up if ≥ 1.5 cm), TR4 – moderately suspicious (FNA if ≥ 1.5 cm, follow up if ≥ 1 cm) and TR5 – highly suspicious (FNA if ≥ 1 cm, follow if ≥ 0.5 cm) [21, 22]. Our patient had TR 2, henceforth FNA was not performed.

THA has been considered a benign congenital anomaly requiring no medical or surgical treatment. Nevertheless, the entity has been associated with a high incidence of other associated thyroid disorders. Therefore, the diagnosis of THA should prompt to investigate for other thyroid disorders for early detection and treatment [1, 7, 21].

## Conclusions

Ultrasound can easily establish the diagnosis of THA and is considered as the investigation of choice. THA is associated with an increased incidence of other thyroid disorders. Therefore, the diagnosis of THA should prompt an active search of other associated morphological or functional thyroid abnormalities. Detection of an associated benign lesion allows a targeted follow-up with patient education and re-assurance.

## Data Availability

The information used and/or analyzed during this case report is available from the corresponding author on reasonable request.
